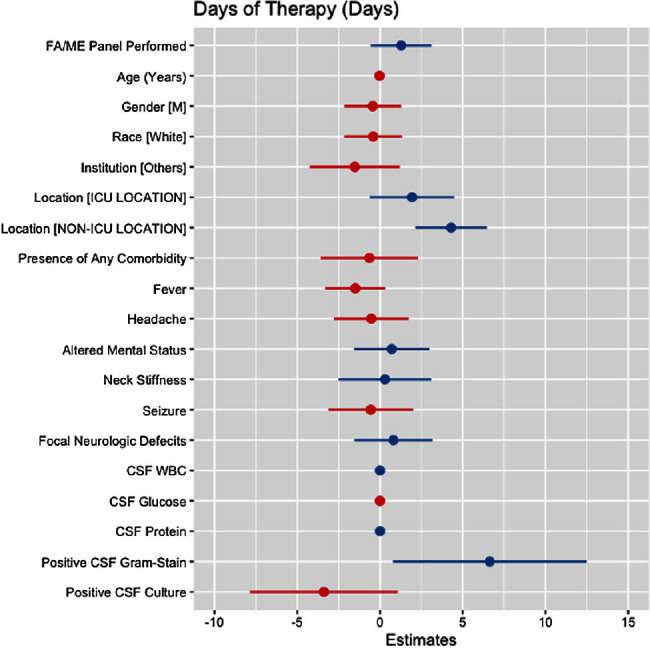# FilmArray Meningitis/Encephalitis Panel Impact on Antibiotic Usage in Patients with Suspected Community-Acquired Meningitis

**DOI:** 10.1017/ash.2024.142

**Published:** 2024-09-16

**Authors:** Aaron Pathak, Caitlynn Pham, Sabra Shay, Todd Lasco, Mayar Al Mohajer

**Affiliations:** Baylor College of Medicine; Premier Inc.

## Abstract

**Background:** Cerebrospinal fluid (CSF) cultures are commonly performed to evaluate patients with suspected bacterial meningitis. These cultures, however, can take up to 72 hours, leading to delays in antibiotic de-escalation and increased antimicrobial utilization. The turnaround for the BioFire® FilmArray® Meningitis/Encephalitis(FA/ME) panel is less than an hour, which may facilitate early de-escalation. Our study aimed to assess whether the use of FA/ME panels in combination with CSF cultures could impact antimicrobial therapy compared to cultures alone in patients treated for suspected bacterial meningitis. **Methods:** Our retrospective study included patients from five hospitals in Texas (2017-2023) who received empiric antibiotics for suspected community-acquired meningitis and underwent a lumbar puncture within 96 hours of admission. Patients with ventricular drains, traumatic brain injury, and non-central nervous system infections were excluded. Cases comprised patients who had an FA/ME panel performed, while controls included patients without the panel. Outcomes were defined as the empiric duration of antimicrobial therapy (days) and total days of antibiotic therapy (DOT). Wilcoxon Rank Sum test and multiple linear regression models were applied to assess the relationship between the use of the FA/ME panel and study outcomes. Independent variables comprised demographics, institution type, acuity, clinical presentation, CSF values, and FA/ME panel use. Imputation was performed using multiple imputation by chained equations. **Results:** A total of 193 patients were included in our study. Seventy-one patients received the FA/ME panel (along with the CSF culture), while 122 patients received the CSF culture alone (controls). The median empiric duration of antibiotic therapy in the cases and controls were 1.71 days and 1.18 days, respectively (p = .160). The median DOT in the cases and controls were eight days and six days, respectively (p = .045). After adjusting for confounders, the FA/ME panel was not significantly associated with changes in the empiric duration of antibiotic therapy (B= 0.18, p = .669, Figure 1) or DOT (B= 1.28, p = .170, Figure 2).

**Conclusion:** Providing FA/ME panel testing without active antimicrobial stewardship interventions did not result in a change in antimicrobial prescribing patterns. The difference from prior literature could be explained by the smaller sample size, limiting the power of the study. Further prospective antimicrobial stewardship efforts should focus on training providers on interpreting FA/ME panel results and providing prospective audits and feedback.

**Figure:**
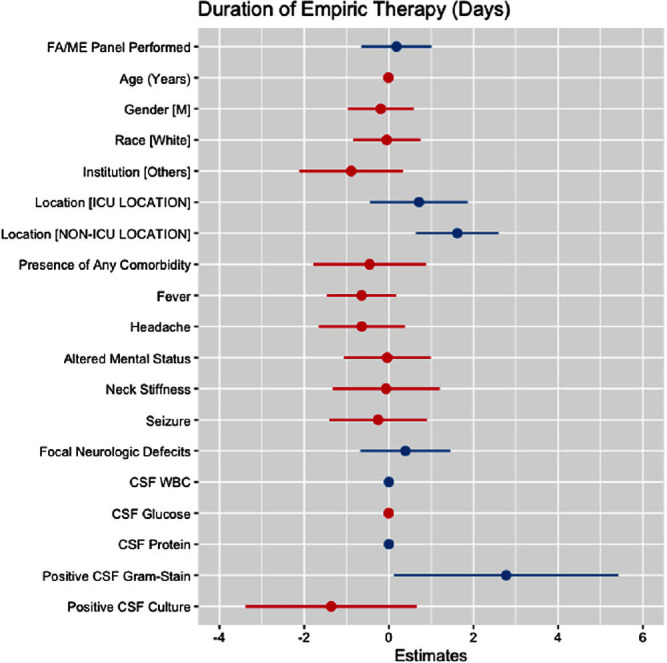

**Figure:**